# Inborn errors of immunity with kidney and urinary tract disorders: a review

**DOI:** 10.1007/s11255-023-03907-4

**Published:** 2024-01-10

**Authors:** Ahmad Shajari, Atefe Zare Ahmadabadi, Mohammad Moein Ashrafi, Tolue Mahdavi, Mahbubeh Mirzaee, Masoumeh Mohkam, Samin Sharafian, Mehrdad Tamiji, Mahnaz Jamee

**Affiliations:** 1 Department of Pediatric Nephrology, Islamic Azad University of Yazd, Yazd, Iran; 2 Immunology and Allergy Department, Mofid Children’s Hospital, Shahid Beheshti University of Medical Sciences, Tehran, Iran; 3 Department of Allergy and Clinical Immunology, Rasool E Akram Hospital, Iran University of Medical Sciences, Tehran, Iran; 4 Pediatric Nephrology Research Center, Research Institute for Children’s Health, Shahid Beheshti University of Medical Sciences, Tehran, Iran; 5 Department of Computer Science, Rice University, Houston, TX USA; 6 Laboratory for Pediatric Immunology, Department of Pediatrics, Willem-Alexander Children’s Hospital, Leiden University Medical Center, Leiden, Netherlands

**Keywords:** Kidney, Renal disorder, Nephropathy, Inborn error of immunity, Primary immunodeficiency

## Abstract

Human inborn errors of immunity (IEIs), previously referred to as primary immunodeficiency disorders (PIDs), are a heterogeneous spectrum of inherited abnormalities of the immune system with different organ involvement. The number of identified IEIs is rapidly increasing, highlighting the non-negligible role of an interdisciplinary approach in clinical diagnosis. Kidney disorders are one of the important comorbidities in some of the affected patients and play a significant role in the diagnosis and course of disease. According to recent studies, 22 types of human IEI with renal manifestations have been identified so far, including immunodeficiency with congenital thrombocytopenia, thymic defects with additional congenital anomalies, complement deficiencies, type 1 interferonopathies, immunity related to non-hematopoietic tissues, congenital neutropenia’s, common variable immunodeficiency disorder (CVID) phenotype and immuno-osseous dysplasia. Based on this classification, we herein review IEIs with renal features and explain the genetic defect, inheritance, and type of renal manifestations.

## Introduction

Inborn errors of immunity (IEI) comprise a group of 485 inherited disorders. Damage to the germinal variants of genes is the cause of these hereditary disorders (1). Indeed, deletion or reduction of the protein expression or function (null/hypomorphic) or protein modification to acquire gain-of-function (GOF) result in altering the encoded gene product and cause IEI, on this basis they divided into dominant or recessive, autosomal, or X-linked, and with complete or incomplete penetrance. IEIs are clinically manifested with a wide spectrum of mild to severe symptoms, including recurrent infections, autoimmunity and autoinflammatory diseases, allergy, bone marrow failure, and often malignancy. Manifestations are related to variant type and its inheritance (2). Although IEIs are considered rare disorders and affect 1 in every 10,000 to 50,000 births based on the type, the recognition of new phenotypes can change its prevalence as the estimated prevalence rate is 1 in every 1000 to 10,000 births [3]. In the past few years, several novel gene defects have been found, and more case reports of these patients have been published [2, 4]. In a number of these studies, the role of kidney disorders in the onset, duration, and end of the disease was reported, and the main cause of death in some of them was related to kidney disease [5–8]. In this study, we review kidney disorders among these patients and discuss the related renal manifestations. To identify relevant articles, we performed an advanced search in PubMed, using the following combination of keywords; nephropathy, renal disorder, renal abnormality, proteinuria, nephrocalcinosis, glomerulonephritis, nephrotic syndrome, autoimmunity, lupus nephritis, hemolytic uremic syndrome (HUS) and Inborn errors of immunity. Based on recent studies 22 types of IEI were described to have kidney disorders as their manifestations, which are listed in Table 1 according to the type of mutation, IEI classification, and the type of kidney disease.

**Table 1:** Inborn errors of immunity with renal features

No	Disease	Genetic defect	Inheritance	Renal manifestations
Complement deficiencies				
1	C3 deficiency (LOF)	*C3*	AR	Glomerulonephritis- hemolytic-uremic syndrome with GOF mutations
2	C3 GOF	*C3*	AD GOF	Atypical hemolytic-uremic syndrome
3	Factor B GOF	*CFB*	AD GOF	Atypical hemolytic-uremic syndrome
4	Factor I deficiency	*CFI*	AR	atypical Hemolytic-uremic syndrome
5	Factor H deficiency	*CFH*	AR or AD	atypical Hemolytic-uremic syndrome
6	Factor H-related protein deficiencies	*CFHR1- CFHR2- CFHR3- CFHR4- CFHR5*	AR or AD	Older onset atypical hemolytic-uremic syndrome
7	Thrombomodulin deficiency	*THBD*	AD	Atypical hemolytic-uremic syndrome
8	Membrane cofactor protein (CD46) deficiency	*CD46*	AD	Atypical hemolytic-uremic syndrome
Common variable immunodeficiency				
9	CD19 deficiency	*CD19*	AR	glomerulonephritis
10	CD81 deficiency	*CD81*	AR	glomerulonephritis
11	RAC2 deficiency	*RAC2*	AR	poststreptococcal glomerulonephritis
Defects in intrinsic and innate immunity				
12	VPS45 deficiency (SCN5)	*VPS45*	AR	Nephromegaly
13	Isolated congenital asplenia (ICA)	*HMOX*	AR	Nephritis
Autoinflammatory disorders				
14	Pediatric systemic lupus erythematosus due to DNASE1L3 deficiency	*DNASE1L3*	AR	lupus nephritis
Combined immunodeficiency				
15	Wiskott–Aldrich syndrome (WAS LOF)	*WAS*	XL	IgA nephropathy
16	Interleukin 6 cytokine family signal transducer	*IL6ST*	AR	Renal abnormalities
17	Sphingosine phosphate lyase insufficiency syndrome (SPLIS)	SGPL1	AR	Nephrotic syndrome
18	Schimke Immuno-Osseous Dysplasia	*SMARCAL1*	AR	Nephropathy
19	Chromosome 10p13-p14 deletion syndrome (10p13-p14DS)	Del10p13-p14	AD	Renal disease
Primary immune regulatory disorders				
20	Autoimmune polyendocrinopathy-candidiasis-Eectodermal dystrophy (APCED)	*AIRE*	AR	Nephrocalcinosis
21	Immune Dysregulation, Polyendocrinopathy, Enteropathy, X-linked (IPEX)	*FOXP3*	XL	Renal failure, nephrotic syndrome, Membranous glomerulonephritis
22	PRKCD deficiency	*PRKCD*	AR	SLE-like autoimmunity (nephrotic and antiphospholipid syndromes)

*AR* autosomal recessive, *GOF* gain-of-function, *LOF* loss-of-function, *AD* autosomal dominant, *CVID* common variable immunodeficiency, *XL* X-linked, AIRE; autoimmune regulator, *SLE* systemic lupus erythematosus

## IEIs with renal manifestations

IEIs are classified into 10 groups in the last update of The International Union of Immunological Societies (IUIS) in 2022 [1], and in this study, most renal manifestations were related to the complements. In Table 1, these diseases are given and in the section below, diseases with more evidence in the literature were reviewed.

### Complement deficiencies

#### Factor H-related protein deficiencies

The five Complement Factor H-related proteins (FHR1–5) are structurally related to factor H, an important regulator of complement alternative pathways [9]. Mutations in FHR by creating duplicated dimerization domains lead to C3 glomerulopathy (C3G), which is a heterogeneous group of chronic kidney diseases [10]. Renner et al. (2022) In a study on murine, reported that variation in genes or expression of FHRs can be associated with glomerular damage by disrupting the regulation of complement binding to mesangial, glomerular endothelial, podocytes, and tubular epithelial cells [11]. Medjeral-Thomas et al. [12] and Zhu et al. [13] identified the increased level of FHR-5 in patients with Immunoglobulin A nephropathy (IgAN) as a risk factor for disease progression.

#### Thrombomodulin deficiency

Thrombomodulin is a membrane protein and cofactor for thrombin which has a strategic role in coagulation, innate immunity and complement regulation [14]. This protein is encoded by the *THBD* gene and variants including A43T (Impaired binding of Complement factor H (CFH) and C3b) [15], P495S and P501L (Moderate reduction of thrombomodulin expression on the cell surface) [16] and D486Y (Less common in patients with venous thrombosis) [17] are identified. It seems that, these variants can cause endothelial damage and microvascular thrombosis and manifest atypical hemolytic-uremic syndrome (aHUS) clinically. Microangiopathic hemolytic anemia in association with thrombocytopenia and acute kidney injury are features of typical aHU. Raina et al. (2022) in a systematic review showed that C3; CH50; AH50; and CFB were lower in aHUS as compared with the reference range [18].

#### Membrane cofactor protein (CD46) deficiency

It has been shown that deficiency in soluble or membrane-bound proteins of the complement system is mainly associated with systemic lupus erythematosus, infection, aHUS, and angioedema [19]. Membrane cofactor protein (MCP; CD46) is an inhibitor of complement activation, whose role in immune complex syndromes has recently been noticed [20]. This protein is involved in inactivating C3b and C4b by serine protease factor I (FI). More than 80 mutations associated with aHUS phenotype are identified in MCP [21–24], accounting for ∼10 − 15% of aHUS cases [25]. It seems necessary to perform related genetic evaluations in children with HUS who develop kidney failure.

### Common variable immunodeficiency (CVID)

#### CD19 & CD81 deficiency

Mutation in *CD19* gene causes symptoms similar to common variable immunodeficiency disorder (CVID) due to strong reduced in antibody production [26]. Recent case-reports presented kidney involvement in children with CD19 defect in the early years of their lives. Primary manifestations may include recurrent hematuria or meningitis and frequent respiratory infections as a result of antibody response to vaccination [26]. Findings such as IgA, IgG and IgM deficiencies and a kidney biopsy result of endocapillary proliferative glomerulonephritis and intense mesangial IgA deposits, can indicate IgA nephropathy in these patients [26]. It seems IgA nephropathy might not be attributable directly to CD19 deficiency since its functions accompany with CD21, CD81, and CD225 and they may also play a significant role [27–29]. Further investigations are needed in this field.

#### RAC2 deficiency

The large family of guanosine triphosphatases (GTPase) enzymes are involved in the hydrolysis of GTP nucleotides to guanosine diphosphate (GDP). The Rac family consists of Rac1, Rac2 (GTPase, exclusively expressed in hematopoietic cells), and Rac3, RhoG, and Rac associated GTPase. Rac2 is the major isoform in human neutrophils that regulate them in neutrophil chemotaxis [6, 30]. RAC2 deficiency impact on wide spectrum of immunity system such as innate arm and B- and T-cell migration, activation, development. Indeed, genetic defect in *RAC2* gene can be associated with a wide range of immune symptoms such as soft-tissue infections which presented few weeks after birth [30]. W56X, D57N, P34H, E62K, N92T, G12R variants have been reported in the Rac2 subset [31]. Alkhairyet et al. reported in 2014 the homozygous loss-of-function mutation in the RAC2 gene, (W56X) causes: CVID, glomerulonephritis, coagulopathy, multiple hormone deficiencies potentially on the autoimmune basis and abnormalities of neutrophil granules [32]. Among patients reported to date due to *RAC2* mutations [5, 33], recurrent sinopulmonary infections was the major clinical manifestation during their first admission. Despite the presence of various types in these patients, only 2 family cases (sister/brother) with W56X diagnosed with post streptococcal glomerulonephritis (PSGN) [34].

#### Sphingosine phosphate lyase insufficiency (SPLIS)

Sphingosine phosphate lyase insufficiency syndrome (SPLIS), caused by defect in *SGPL1* gene, is a childhood syndrome that mainly manifests with steroid-resistant nephrotic syndrome (SRNS), primary adrenal insufficiency, rapid or insidious neurologic deterioration, immunodeficiency, and acanthosis [35]. Sphingosine-1-phosphate lyase (SPL) enzyme is involved in irreversible degradation of sphingolipid into phosphoethanolamine and hexadecimal and its removal [36]. Kidney involvement mainly manifests as steroid-resistant nephrotic syndrome, which can be congenital or occur during infancy, may progress into end-stage kidney disease in the first year of life, and seems to be correlated with a higher mortality rate [37]. In these patients, nephrotic syndrome may be the primary or isolated manifestation, and the outcome of kidney transplantation is largely unknown [38].

## Combined immunodeficiency (CID)

### Wiskott—Aldrich syndrome (WAS)

X-linked Wiskott–Aldrich syndrome (WAS), first recognized in 1937 by Dr. Alfred Wiskott, and then in 1957, confirmed by Robert Aldrich [39].it is estimated to involve 1–10 live births per million males. The *WAS* gene encodes a cytosolic protein known as WAS protein (WASp) and it expressed in myeloid, lymphoid, and hematopoietic stem cells (HSCs), which can lead to various clinical manifestations, but thrombocytopenia (X-linked thrombocytopenia [XLT]), eczema, and recurrent infections with increased risk of lymphoid malignancies, autoimmune disorders or congenital neutropenia (X-lined neutropenia [XLN]) are classic phenotype of WAS [40, 41]. The patients with thrombocytopenia show a different range of symptoms from a mild petechiae and purpura to serious intracranial and/or intestinal bleeding, which lead to death in 4–10%. The mean platelet volume (MPV) in WAS is often < 5 fL, so it can reach < 2 fL in the patients with severe bleeding symptoms. In addition, this disease is usually misdiagnosed with immune thrombocytopenia (ITP), and it should be noted that the WAS hallmark is micro thrombocytopenia (small platelets) [41]. Eczema can affect 75% of these patients, and usually shows itself as refractory and the patient is not able to treat it quickly [42]. Recent studies reported renal involvements including IgA nephropathy (IgAN), membranoproliferative glomerulonephritis (MPGN) and interstitial nephritis in 3.5–19% of the patients, often progressing to renal failure and require transplantation [40, 43–45]. Although various studies have investigated the role of different markers in the occurrence of renal complications in WAS, no specific etiology has been elucidated [46–49].

#### Interleukin 6 cytokine family signal transducer

Interleukin-6 signal transducer (IL6ST) encodes the GP130 protein. In pro-inflammatory conditions, GP130 signaling IL6 cytokine. IL-6 family cytokines are involved in the regulation of the hepatic acute-phase reaction, in B-cell stimulation, in the regulation of the balance between regulatory and effector T cells, in metabolic regulation, and in many neural functions [50]. Biallelic loss-of-function IL6ST variants cause autosomal recessive hyper-IgE syndrome or a variant of the Stuve–Wiedemann syndrome (skeletal dysplasia, lung dysfunction, congenital thrombocytopenia, dermatitis, renal abnormalities, and defective acute-phase response) [34]. Chen et al. (2020) reported a Saudi Arabic family with two fetuses with mutations in IL6S and were homozygous for c.841C > T; p.Arg281*.The first fetus had short femora (less than fifth centile), bowed tibia, multicystic dysplastic right kidney, dilated cisterna magna, and a small chest and died 3 h after delivery [34].

#### Schimke immuno-osseous dysplasia

Schimke Immuno-Osseous Dysplasia (SIOD) is an autosomal recessive multi-system disorder, with the genetic cause of biallelic loss-of-function mutation in *SMARCAL1* (SWI/SNF-related, matrix-associated, actin-dependent regulator of chromatin, subfamily a-like 1) gene [8]. The incidence rate of SIOD is 1 per 1–3 million live births and is clinically manifested by growth failure, spondyloepiphyseal dysplasia, lymphopenia, and proteinuria. Hypothyroidism, cerebral ischemia, and bone marrow failure were also reported in half of these patients [7]. Nephropathy in SIOD children is described as progressive and steroid-resistant [51]. The pathophysiology of nephropathy in SIOD remained unknown but the role of the *SMARCAL1* gene in the renal progenitor population is suggested in the study of Dekel et al. [52]. In this study, according to the neonatal/adult murine kidney, it is explained that peak levels of SMARCAL1 and localization in glomerular podocytes can explain proteinuria and progressive nephropathy in SIOD children.

## Type-1 interferonopathies

### Pediatric systemic lupus erythematosus due to DNASE1L3 deficiency

The prevalence and incidence of lupus in children is not precisely known due to different age definitions [35]. But since the role of gender and sex hormones in the occurrence of lupus is less prominent at a young age, the role of genetics in this field becomes more prominent. The disease usually involves skin, musculoskeletal system, and kidneys, of which the latter is the determinative manifestation for prognosis [53]. Al-Mayouf et al. (2011) investigated six families with pediatric lupus and found a role for DNASE1L3 loss-of-function mutations in these patients [36]. Loss-of-function mutations in DNASE1L3 cause high disease activity with variable degrees of renal involvement in children. Also, In the study by Kisla et al. (2021) a child with DNASE1L3 deficiency who suffered from urticarial skin lesions, frequent hemoptysis, and kidney involvement, was finally diagnosed as this rare monogenic lupus [53].

## Primary immune regulatory disorders

### Immune dysregulation, polyendocrinopathy, enteropathy, X-linked syndrome

The significant number of new cases of this syndrome indicates that Immune Dysregulation, Polyendocrinopathy, Enteropathy, X-linked Syndrome (IPEX) is a rare disease that is underestimated. IPEX is characterized by immune dysregulation, polyendocrinopathy, enteropathy, and an X-linked inheritance pattern. Mutations related to the fork head box p3 (FOXP3) transcription factor, which is the main gene of regulatory T-cells (Treg), are involved in the development of this disease in interaction with environmental factors. Various studies have investigated the clinical manifestations of these patients. Some of These manifestations include autoimmune enteropathy, Type-1 diabetes mellitus, Dermatitis, and frequent infections. In the study of Rao et al. [54] (2007) and Moudgil et al. [55] (2007), renal involvement in these patients was reported as Membranous glomerulonephritis (MGN). Other reported kidney involvement in these patients were Renal failure, tubulointerstitial nephritis (TIN) and nephrotic syndrome [56].

### Autoimmune polyendocrinopathy-candidiasis-ectodermal dystrophy (APECED)

Mutations in the gene encoding immune regulatory (AIRE) protein cause autoimmune polyendocrinopathy-candidiasis-ectodermal dystrophy (APECED) with recessive or dominant autosomal inheritance. In this syndrome, the patient suffers from chronic Candida infection in childhood and then autoimmune hypoparathyroidism and autoimmune primary adrenocortical failure [57].If there are two of these three components or a previous diagnosis in the patient's sibling, this disease is raised. Tissue-specific antibodies and cytokines created during this syndrome can rarely cause kidney involvement and autoimmune TIN [58]. The symptoms of TIN are non-specific and sometimes less clear, but unexplained fever is one of its notable features [59]. Increased creatinine, ESR, abnormal urinalysis, proteinuria or decreased kidney function without abnormalities in urinalysis can be a clue to suspect this syndrome in susceptible patients (58).

## Conclusion

**Fig. 1:**
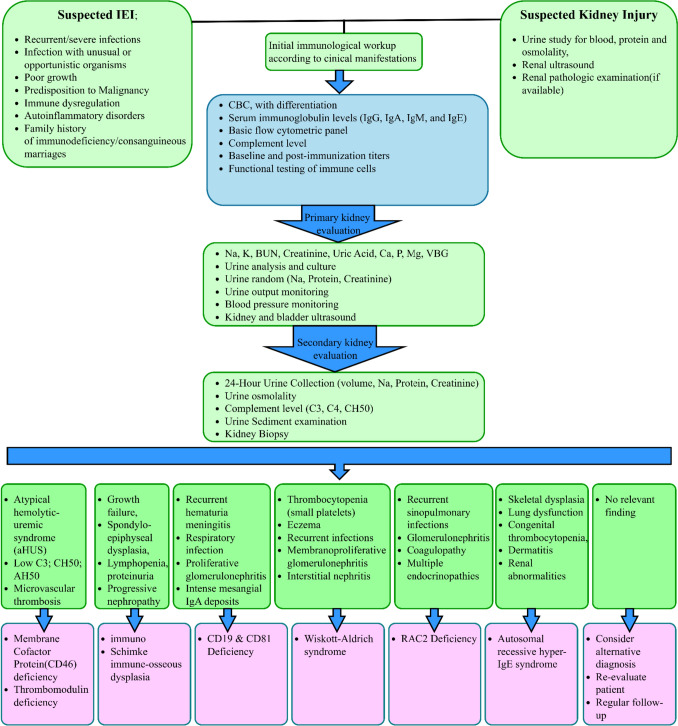
Step-wise approach for evaluation of kidney and urinary tract disorders in patients with inborn errors of immunity

Inborn errors of immunity are multi-system disorders and require interdisciplinary approach for optimal diagnosis and management. Among others, the characteristics of kidney and urinary system in IEIs have remained obscure and need more consideration by the pediatrics, nephrologists and immunologists. As illustrated in Fig. 1, a thorough evaluation of kidney and urinary system in patients with abnormal initial renal screening should be performed concomitantly with immunology workup. This evaluation is divided in primary and secondary steps and will be helpful in earlier clinical diagnosis of the underlying IEIs before the result of the genetic study become ready. In this review, we tried to summarize the IEIs reported to manifest renal abnormalities, however, the list provided here is according to the well-established IEIs and certainly in the future it will be extended.

## Data Availability

Data available within the article.

## Abbreviations

IEI: Inborn error of immunity
GOF: Gain-of-function
FI: Factor I
CFH: Complement factor H
aHUS: Atypical hemolytic-uremic syndrome
MCP: Membrane cofactor protein
IgAN: Immunoglobulin A nephropathy
SIOD: Schimke Immuno-Osseous Dysplasia
FHR1–5: Factor H-related proteins
WAS: Wiskott–Aldrich syndrome
WASp: WAS protein
HSCs: Hematopoietic stem cells
XLT: X-linked thrombocytopenia
XLN: X-lined neutropenia
MPV: Mean platelet volume
MPGN: Membranoproliferative Glomerulonephritis
CVID: Common variable immunodeficiency disorder
C3G: C3 glomerulopathy
GTPase: Guanosine Triphosphatases
GDP: Guanosine Diphosphate
PSGN: Poststreptococcal glomerulonephritis
JAK/STAT: Janus kinase signal transducers and activators of transcription pathway
IUIS: International Union of Immunological Societies
MGN: Membranous glomerulonephritis
IPEX: Immune Dysregulation, Polyendocrinopathy, Enteropathy, X-linked Syndrome
FOXP3: Fork head box P3
Treg: Regulatory T-cell
SPLIS: Sphingosine phosphate lyase insufficiency syndrome
SRNS: Steroid resistant nephrotic syndrome
SPL: Sphingosine-1-phosphate lyase
